# Stress ulcer prophylaxis versus placebo or no prophylaxis in adult hospitalised acutely ill patients—protocol for a systematic review with meta-analysis and trial sequential analysis

**DOI:** 10.1186/s13643-017-0509-4

**Published:** 2017-06-24

**Authors:** Søren Marker, Anders Perner, Jørn Wetterslev, Marija Barbateskovic, Janus Christian Jakobsen, Mette Krag, Anders Granholm, Carl Thomas Anthon, Morten Hylander Møller

**Affiliations:** 1 Department of Intensive Care, 4131, Copenhagen University Hospital Rigshospitalet, Copenhagen, Denmark; 2 Copenhagen Trial Unit, Centre for Clinical Intervention Research, Copenhagen University Hospital Rigshospitalet, Copenhagen, Denmark; 3 Centre for Research in Intensive Care (CRIC), Copenhagen, Denmark; 4 Department of Cardiology, Holbaek Hospital, Holbaek, Denmark

**Keywords:** Acid suppressants, Gastrointestinal bleeding, Risk factors, Side effects, Stress ulcer prophylaxis, Proton pump inhibitors, Histamine-2-receptor antagonists

## Abstract

**Background:**

Stress ulcer prophylaxis is considered standard of care in many critically ill patients in the intensive care unit (ICU). However, the quality of evidence supporting this has recently been questioned, and clinical equipoise exists. Whether there is overall benefit or harm of stress ulcer prophylaxis in adult hospitalised acutely ill patients is unknown. Accordingly, we aim to assess patient-important benefits and harms of stress ulcer prophylaxis versus placebo or no treatment in adult hospitalised acutely ill patients with high risk of gastrointestinal bleeding irrespective of hospital setting.

**Methods/design:**

We will conduct a systematic review of randomised clinical trials with meta-analysis and trial sequential analysis and assess use of proton pump inhibitors (PPIs) or histamine-2-receptor antagonists (H2RAs) in any dose, formulation and duration. We will accept placebo or no prophylaxis as control interventions. The participants will be adult hospitalised acutely ill patients with high risk of gastrointestinal bleeding.

We will systematically search the Cochrane Library, MEDLINE, EMBASE, Science Citation Index, BIOSIS and Epistemonikos for relevant literature. We will follow the recommendations by the Cochrane Collaboration and the Preferred Reporting Items for Systematic Review and Meta-Analysis (PRISMA) statement. The risk of systematic errors (bias) and random errors will be assessed, and the overall quality of evidence will be evaluated using the Grading of Recommendations Assessment, Development, and Evaluation (GRADE) approach.

**Discussion:**

There is a need for a high-quality systematic review to summarise the benefits and harms of stress ulcer prophylaxis in hospitalised patients to inform practice and future research. Although stress ulcer prophylaxis is used worldwide, no firm evidence for benefit or harm as compared to placebo or no treatments has been established. Critical illness is a continuum not limited to the ICU setting, which is why it is important to assess the benefits and harms of stress ulcer prophylaxis in a wider perspective than exclusively in ICU patients.

**Systematic review registration:**

PROSPERO CRD42017055676

**Electronic supplementary material:**

The online version of this article (doi:10.1186/s13643-017-0509-4) contains supplementary material, which is available to authorized users.

## Background

### Description of the condition

Critically ill patients are at risk of stress-related mucosal damage [1]. After 3 days of stay in the intensive care unit (ICU), the majority of patients have developed endoscopically evident signs of gastrointestinal ulcerations [2, 3]. These stress ulcerations are typically superficial and asymptomatic, but 1 in 20 will progress and erode larger vessels resulting in overt gastrointestinal bleeding [4, 5]. Gastrointestinal bleeding is a potentially life-threatening condition with a mortality of up to 50% among frail patients [6]. However, the reported prevalence of gastrointestinal bleeding varies between 2 and 5% possibly because of heterogeneous populations, varying definitions and difficulties in diagnosing stress ulcers [5, 7–10]. Importantly, the reported estimates often include all conditions resulting in gastrointestinal bleeding and not exclusively stress ulcers and other bleedings prevented by acid suppressants. In a cohort study by Cook et al. [11], stress ulceration was identified as the sole source of gastrointestinal bleeding by endoscopy in less than 50% of the patients, suggesting that other causes of gastrointestinal bleeding, not prevented by stress ulcer prophylaxis, are frequent. This highlights the need for placebo-controlled trials to ensure assessment of stress ulcer-induced bleedings only.

Several studies have sought to identify risk factors for developing gastrointestinal bleeding in critically ill patients. A 1994 landmark study highlighted mechanical ventilation (for >48 h) and coagulopathy as major risk factors for gastrointestinal bleeding [6]. Later studies, however, have not consistently been able to reproduce the association with mechanical ventilation [5, 8]. Recently, acute kidney injury, hepatic failure and acute and chronic disease severity have also been suggested as important risk factors [5, 8, 12].

### Description of the intervention

In order to prevent the potential progression from stress-related mucosal damage to gastrointestinal bleeding, stress ulcer prophylaxis was introduced more than 40 years ago [13]. Initially, antacids and later sucralfate were the preferred agents. The introduction of histamine-2-receptor antagonists (H2RAs) made intravenous administration possible. A randomised clinical trial (RCT) from 1998 by Cook et al. reported a lower incidence of gastrointestinal bleeding in patients receiving H2RA compared with sucralfate [14]. Proton pump inhibitors (PPIs) were introduced later on, and today, the majority of prescribed stress ulcer prophylaxis is H2RAs or PPIs [5, 15].

International guidelines recommend the use of stress ulcer prophylaxis with PPI or H2RA as standard of care in high-risk ICU patients [16]. However, the rationale and level of evidence of stress ulcer prophylaxis in ICU patients has been questioned because of limited data, methodological flaws in some trials, potential increased rates of hospital-acquired pneumonia, *Clostridium difficile* enteritis and myocardial ischemia following the use of stress ulcer prophylaxis, and general improvements in intensive care [1, 17–19].

### How the intervention might work

It has been hypothesised that stress ulcerations are caused by decreased mucosal blood flow, ischemia and reperfusion injury and hence are less related to acid secretion than peptic ulcerations [20]. However, the pathophysiology behind stress ulcerations has not been fully elucidated.

H2RAs inhibit the stimulation of the H^+^-K^+^-adenosine triphosphatase (ATPase) by binding to the H2-receptor on the parietal cells [21]. This results in diminished gastric acid secretion. H2RAs can be administered enterally or intravenously, and continuous intravenous infusion seems to be more effective than bolus injections at controlling gastric pH [22]. PPIs are among the most frequently prescribed drugs in the world [21]. They inhibit secretion of gastric acid by forming irreversible disulfide bonds with the H^+^-K^+^-ATPase pump. This leads to inhibition of the secretion of gastric acid. PPIs can be administered enterally or intravenously, and the irreversible bond provides a stronger and more prolonged reduction of acid secretion compared to H2RAs [21].

### Why it is important to do this review

The effects of PPIs and H2RAs have been compared in several RCTs and meta-analyses [17, 23–26], with the latest indicating that PPIs results in better protection against both clinically important and overt gastrointestinal bleeding compared with H2RAs [26]. However, as neither PPIs nor H2RAs have been found superior to placebo, this might be of questionable clinical relevance. In the most recent systematic review of stress ulcer prophylaxis (PPI or H2RA) versus placebo or no prophylaxis in general ICU patients (20 trials), it was concluded that the quantity and quality of evidence supporting the use of stress ulcer prophylaxis is low with no firm evidence for benefit or harm [27].

Additional trials have [28–30] and may have been published, and it is necessary to include these trial estimates in a meta-analysis to provide an up-to-date assessment on patient-important benefits and harms. Existing evidence on benefits and harms of stress ulcer prophylaxis mainly derives from trials conducted in the ICU [27]. Critical illness is a continuum not limited to the ICU setting, which is why it is important to assess the benefits and harms of stress ulcer prophylaxis in acutely ill patients with high risk of gastrointestinal bleeding not limited to the ICU setting. Inclusion of non-ICU high-risk patients may also add to the questioned role of mechanical ventilation as a trigger of stress ulcers. Despite the clinical equipoise, common beliefs and practices across various medical specialties are that certain subpopulations of acutely ill patients benefit from stress ulcer prophylaxis [31].

The use of stress ulcer prophylaxis is a clinical dilemma. It remains unresolved whether acid suppressants prevent stress-related gastrointestinal bleeding in acutely ill patients. The prevalence of gastrointestinal bleeding is low, and the balance between benefits and harms of stress ulcer prophylaxis is unknown. Whether there is overall benefit or harm of stress ulcer prophylaxis is ambiguous, and to ensure patient safety, there is a need for large, high-quality RCTs of stress ulcer prophylaxis versus placebo and well-designed systematic reviews.

## Objectives

We aim to assess patient-important benefits and harms of stress ulcer prophylaxis versus placebo or no prophylaxis in adult hospitalised acutely ill patients with high risk of gastrointestinal bleeding irrespective of hospital setting.

Research question: does stress ulcer prophylaxis improve patient-important outcomes in adult hospitalised acutely ill patients with high risk of gastrointestinal bleeding as compared with placebo or no prophylaxis?

## Methods

### Study design

We will conduct a systematic review of RCTs with meta-analysis and trial sequential analysis (TSA) and follow the recommendations by the Cochrane Collaboration [32] and the Preferred Reporting Items for Systematic Review and Meta-Analysis (PRISMA) statement [33].

### Study registration

This protocol has been prepared according to the Preferred Reporting Items for Systematic Review and Meta-Analysis Protocols (PRISMA-P) guidelines [34] (checklist included as Additional file 1), and the review has been registered in the International Prospective Register of Systematic Reviews (PROSPERO) (https://www.crd.york.ac.uk/prospero/; registration number CRD42017055676).

### Criteria for considering studies for this review

#### Types of studies

RCTs will be included regardless of publication source, status and language. Quasi-randomised trials and cross-over trials will be excluded.

#### Types of participants

Adult hospitalised acutely ill patients (as defined by the included trials) with high risk of gastrointestinal bleeding including (but not limited to) medical and surgical ICU patients, patients in intermediate care units, patients in coronary care facilities, neurosurgical patients (head and spine), cardiothoracic surgical patients, organ transplanted patients, major abdominal/vascular/orthopaedic (pelvis and hip) surgery, burn injured patients (incl. thermal injury), patients with active malignant haematological illness, patients with acute kidney injury, patients with acute hepatic failure, patients receiving high dose steroids (at least 0.3 mg/kg/day of prednisolone equivalent) and patients with sepsis.

Trials including children (as defined by the original trials) will be excluded unless data can be separately extracted for adults only.

#### Types of interventions

- Experimental intervention: any type of PPI (omeprazole, lansoprazole, dexlansoprazole, esomeprazole, pantoprazole and rabeprazole) or H2RA (nizatidine, famotidine, cimetidine and ranitidine) in any dose, formulation, timing and duration
- Control intervention: placebo or no prophylaxis

### Types of outcome measures

#### Primary outcomes

- All-cause mortality
- Proportion of participants with one or more serious adverse event (SAE), defined as any untoward medical occurrence that resulted in death, was life-threatening or prolonged existing hospitalisation, resulted in persistent or significant disability or any important medical event, which may have jeopardised the patient [35]
- Proportion of participants with ‘clinically important gastrointestinal bleeding’ as defined in the included trials (not including ‘overt GI bleeding’)

#### Secondary outcomes

- Proportion of participants with *C. difficile* enteritis (yes/no), as defined in the included trials
- Proportion of participants with myocardial infarction (yes/no), as defined in the included trials
- Proportion of participants with hospital-acquired pneumonia (yes/no), as defined in the included trials
- Quality-of-life (any continuous scale used in the included trials)

##### Assessment time points

All outcomes will primarily be assessed at the time point closest to 90 days. Secondly, we will assess all outcomes at the maximum time of follow-up.

### Search methods for identification of studies

#### Electronic searches

We will search the following electronic databases:

- Cochrane Library
- MEDLINE
- EMBASE
- Epistemonikos
- Science Citation Index
- BIOSIS

We will additionally search databases of ongoing trials including Clinical-Trials.gov (http://clinicaltrials.gov/), *meta*Register of Controlled Trials (http://www.isrctn.com/page/mrct), the EU Clinical Trials register (https://www.clinicaltrialsregister.eu/) and the World Health Organization (WHO) International Clinical Trials Registry Platform Search Portal (http://apps.who.int/trialsearch/).

The tentative Medline search strategy is available in Additional file 2. To continuously identify newly published studies, we will apply PubMed’s ‘My NCBI’ (National Center for Biotechnology Information) email alert service. Before we submit the final review draft to an international peer-reviewed journal, we will perform an updated search on all specified databases. If we identify new trials, these will be assessed and if relevant incorporated in our review before submission of the final review draft. If we detect additional relevant keywords during any of the electronic or other searches, we will modify the electronic search strategies to incorporate these terms and document the changes.

#### Searching other resources

We will hand-search the reference list of relevant trials and other systematic reviews and meta-analyses on stress ulcer prophylaxis in adult hospitalised acutely ill patients.

Unpublished trials will be sought identified. Authors will be contacted for additional data if relevant.

### Data collection and analysis

#### Selection of studies

Two review authors (SM, AG or CTA) will independently screen the abstract, title or both, of every record retrieved, to determine which trials should be assessed further. We will assess all potentially relevant articles as full text. We will resolve any discrepancies through consensus or recourse to a third review author (MHM). If resolving disagreement is not possible, the article will be added to those ‘awaiting assessment’ and we will contact study authors for clarification. We will present an adapted PRISMA flowchart of study selection [33]; see Additional file 3.

#### Data extraction and management

Two review authors (SM, AG or CTA) will independently extract information from each included trial using a predefined data extraction form. The extracted information will include trial characteristics (year of publication, duration, country), characteristics of the trial participants (inclusion criteria and exclusion criteria), type of intervention/control (name, dosing, duration and route of administration), type of control (name, dosing, duration and route of administration), outcomes and risk of bias.

In the event of duplicate publications, companion documents or multiple reports of a primary study, we will maximise yield of information by comparing all available data and use the most complete dataset aggregated across all known publications. In case of doubt, the publication reporting the longest follow-up associated with our primary or secondary outcomes will be given priority.

#### Assessment of risk of bias in included studies

We will assess the risk of systematic errors (bias) of the included trials according to the Cochrane Handbook [32]. The assessment will be performed independently by two authors (SM, AG or CA). Disagreements will be resolved by consensus upon consultation with a third author (MHM).

The following domains will be assessed: (1) random sequence generation, (2) allocation concealment, (3) blinding of participants and personnel, (4) blinding of outcome assessment, (5) incomplete outcome data, (6) selective reporting and (7) other bias, including baseline imbalance, early stopping and bias due to vested financial interest or academic bias. If one or more domains are judged as being high or unclear, we will classify the trial as having overall high risk of bias [32]. We will assess the domains ‘blinding of outcome assessment’, ‘incomplete outcome data’ and ‘selective outcome reporting’ for each outcome. Thus, we will be able to assess the bias risk for each result in addition to each trial.

We will base our primary conclusions as well as our presentation in the ‘Summary of findings table’ section on the results of our primary outcomes with low risk of bias.

The risk of bias will be depicted in a ‘risk of bias summary’ figure, reviewing the authors judgements (according to the Cochrane handbook [32]) about each included risk of bias item for each included study (*red*: high risk, *green*: low risk, *yellow*: unclear).

#### Measures of treatment effect

We will calculate relative risks (RRs) with 95% confidence intervals (CIs) and mean differences (MDs) with 95% CIs for dichotomous outcomes and continuous outcomes, respectively.

#### Dealing with missing data

The relevant authors will be sought contacted for missing outcome data. Sensitivity analysis using imputations of missing outcome data of dichotomous outcomes in best-worse and worse-best case scenarios will be performed assuming:

1. All patients lost to outcome assessment (follow-up) in the intervention group did not experience the outcome of interest, while all patients lost to outcome assessment (follow-up) in the control group did experience the outcome of interest.
2. All patients lost to outcome assessment (follow-up) in the intervention group did experience the outcome of interest, while all patients lost to outcome assessment (follow-up) in the control group did not experience the outcome of interest.

When analysing continuous outcomes with missing data, we will use imputations of missing outcome data in best-worse and worse-best case scenarios [36] assuming:

1. All patients lost to outcome assessment (follow-up) in the intervention group have an outcome being the group mean plus two standard deviations (SDs) of the group mean, and all patients lost to outcome assessment (follow-up) in the control group will be the group mean minus two SDs.
2. All patients lost to outcome assessment (follow-up) in the intervention group have an outcome being the group mean minus two SDs of the group mean, and all patients lost to outcome assessment (follow-up) in the control group will be the group mean plus two SDs.

#### Assessment of heterogeneity

Based on a previous systematic review and meta-analysis where statistical and clinical heterogeneity was limited, we plan to report pooled effect estimates [27]. We will primarily inspect forest plot for signs of statistical heterogeneity. We will secondly use *D*-squared and *I*-squared statistics to describe heterogeneity among the included trials. We will use and report a fixed-effect model if *I*-squared = 0 and use and report the results of both random-effects model and fixed-effect model if *I*-squared >0. We will report the most conservative estimate if the intervention effects differ in the two models and the broadest confidence interval if they concur [36].

#### Assessment of reporting bias

We will use a funnel plot to assess reporting bias if ten or more trials are included. For dichotomous outcomes, we will test asymmetry with the Harbord test [37]. For continuous outcomes, we will use the regression asymmetry test [38] and the adjusted rank correlation [39].

#### Data synthesis

We will use Review Manager Software (RevMan 5.3) as statistical software.

We will calculate summary estimates (conventional meta-analyses) as outlined above. We will use and report results based on the analysis of intention-to-treat populations if available.

#### Trial sequential analysis

We will conduct TSA in order to assess the risk of random errors [40–42]. Cumulative meta-analyses are at risk of producing random errors due to sparse data and multiple testing of accumulating data [32, 43–50]. TSA allows to estimate the required information size (the number of participants) needed to detect or reject an a priori pre-specified realistic intervention effect in a meta-analysis and the TSA-adjusted CIs [51, 52]. The required information size will take into account the event proportion in the control group, the assumption of a plausible RR reduction and the heterogeneity variance of the meta-analysis [52, 53]. We will use conservative estimations of the anticipated intervention effect estimates to reduce the risk of random error [36]. In brief, as we have three co-primary outcomes and four secondary outcomes, we will consider a *P* < 0.025 and *P* < 0.020 as statistically significant, respectively [35].

We will apply trial sequential monitoring boundaries according to an a priori 15% relative risk difference (reduction or increase), with a family-wise error rate (FWER) equal to an alfa of 5%, beta 90% and a control event proportion suggested by all the trials reporting the outcome in question. TSA-adjusted CIs will be provided [42].

#### Subgroup analysis and investigation of heterogeneity

We will use Chi-squared test to provide an indication of heterogeneity between trials, with *P* = 0.10 considered significant.

We plan to conduct the following subgroup analyses:

1. Comparing estimates of the pooled intervention effect in trials with overall low risk of bias vs. overall high risk of bias. Hypothesised direction of sub-group effect: increased beneficial intervention effect in the trials with overall high risk of bias
2. Comparing estimates of the pooled intervention effect in trials using PPI vs. H2RA. Hypothesised direction of sub-group effect: increased beneficial intervention effect in trials using PPI
3. Comparing estimates of the pooled intervention effect in trials using placebo vs. no treatment. Hypothesised direction of sub-group effect: increased beneficial intervention effect in trials using no treatment
4. Comparing estimates of the pooled intervention effect in the included subpopulations of adult hospitalised acutely ill patients. Hypothesised direction of sub-group effect: increased beneficial intervention effect in some subpopulations
5. Comparing estimates of the pooled intervention effect in ICU patients vs. non-ICU patients. Hypothesised direction of sub-group effect: increased beneficial intervention effect in ICU patients vs. non-ICU patients

#### Sensitivity analysis

We will conduct sensitivity analysis by performing empirical continuity adjustments in the zero event trials.

#### Summary of findings table

We will assess the overall quality of evidence for each outcome measure according to the Grading of Recommendations Assessment, Development, and Evaluation (GRADE) approach [54]. In brief, we will downgrade the quality of evidence (our confidence in the effect-estimates) for an intervention for identified risks of bias, inconsistency (unexplained heterogeneity), indirectness (including other patient populations or use of surrogate outcomes), imprecision (wide confidence interval around the effect estimate) and publication bias. Accordingly, the overall quality of evidence will be rated ‘high’, ‘moderate’, ‘low’ or ‘very low’.

## Discussion

This systematic review will provide updated important knowledge on use of stress ulcer prophylaxis in adult hospitalised acutely ill patients irrespective of hospital setting. We will include recently published RCTs and include and assess adult hospitalised acutely ill patients with high risk of gastrointestinal bleeding both in- and outside the context of the ICU.

New RCTs may have provided important new data on the potential harm of stress ulcer prophylaxis, including the risk of cardiovascular events, hospital-acquired pneumonia and *C. difficile* infections, which is why an updated systematic review is warranted.

As critical illness is not restricted to ICU patients, we believe a wider scope is mandated in order to more accurately evaluate the attempted prevention of gastrointestinal bleedings due to stress ulcers. In a recent cohort study [5], the incidence of gastrointestinal bleedings peaked on the first 1–2 days of ICU stay, suggesting that the risk of developing stress ulcerations and gastrointestinal bleeding in acutely ill patients is not limited to the ICU setting, but is a continuum not defined by the clinical setting. Consequently, an assessment of benefits and harms of stress ulcer prophylaxis in high-risk patients irrespective of clinical setting is needed.

Using TSA to assess the risk of random errors will increase the validity of the summary estimates calculated. The risk of type I errors (a false positive finding) are increased when multiple testing is done, e.g. when analysing multiple primary and secondary outcomes, during repeated testing of data, and, importantly, when meta-analyses are conducted and updated [46]. Also, the risk of type II errors (a false negative finding) challenge the results of meta-analyses, due to sparse data. Statistically significant meta-analyses with few participants have low reliability, and the interventional effect is often overrated [45, 53]. Furthermore, TSA allows to estimate if the required information size (sample size) has been reached and if additional trials are needed [45, 46, 49, 50].

Finally, this systematic review may aid data monitoring and safety committees when assessing results of interim analyses of ongoing and upcoming trials on stress ulcer prophylaxis in acutely ill patients (SUP-ICU [55], REVISE [56], PEPTIC [57]). A modern data monitoring and safety committee cannot allow themselves to conclude solely on the data provided from the interim-analysis within a specific single trial as evidence from the ‘outside’ may influence the overall decision of whether the trial shall continue or not. Therefore, to properly summarise the evidence provided before and during the trial, the data monitoring and safety committee must include the interim-part of the trial data in meta-analyses of all the evidence provided so far using e.g. TSA to reach realistic and firm results for the actual achieved evidence.

Today, stress ulcer prophylaxis with PPI or H2RA is recommended in international guidelines and regarded as standard of care in adult ICU patients [16]. However, the quantity and quality of evidence supporting use of stress ulcer prophylaxis in ICU patients is low with no firm evidence for benefit or harm, and clinical equipoise exist [1, 17–20]. The outlined systematic review will provide important data on the benefits and harms of stress ulcer prophylaxis in adult hospitalised acutely ill patients with high risk of gastrointestinal bleeding in and outside the context of the ICU.

## Additional files

Additional file 1: Checklist of PRISMA-P recommendations. (DOCX 36 kb)

Additional file 2: Tentative search strategy for MEDLINE database. (DOCX 21 kb)

Additional file 3: PRISMA flowchart of study selection. (DOCX 39 kb)

## Abbreviations

AKI: Acute kidney injury
ATPase: Adenosine Triphosphatase
CI: Confidence interval
FWER: Family wise error rate
H2RA: Histamine-2-receptor antagonist
ICU: Intensive care unit
MD: Mean difference
NCBI: National Center for Biotechnology Information
PPI: Proton pump inhibitor
PRISMA-P: Preferred Reporting Items for Systematic Review and Meta-Analysis Protocols
PROSPERO: International Prospective Register of Systematic Reviews
RCT: Randomised clinical trial
RR: Relative risk
SAE: Serious adverse event
SD: Standard deviation
SUP-ICU: Stress Ulcer Prophylaxis in the Intensive Care Unit
TSA: Trial sequential analysis
WHO: World Health Organization
