# Supplementary material for: Stress ulcer prophylaxis versus placebo or no prophylaxis in adult hospitalised acutely ill patients—protocol for a systematic review with meta-analysis and trial sequential analysis
Source: Syst Rev. 2017 Jun 24;6:118. doi: 10.1186/s13643-017-0509-4 (PMC5483291; doi:10.1186/s13643-017-0509-4)
Supplement: Supplementary file 2 — Tentative search strategy for MEDLINE database. (DOCX 21 kb) [file 13643_2017_509_MOESM2_ESM.docx]

**Draft for MEDLINE search strategy**

**MEDLINE - 17.01.17**

1. exp Peptic Ulcer/dt, pc, th [Drug Therapy, Prevention & Control, Therapy]
2. exp Gastrointestinal Hemorrhage/dt, pc, th [Drug Therapy, Prevention & Control, Therapy]
3. ((stress or stomach or peptic) adj2 ulcer).mp.
4. gastrointestinal bleeding.mp.
5. exp Proton Pumps/
6. exp Proton Pump Inhibitors/
7. (PPI or PPIs or (proton adj3 pump adj3 inhibitor$)).mp.
8. (dexlansoprazole or kapidex or dexilant).mp.
9. (esomeprazole or nexium or esotrex or alenia or escz or esofag or nexiam).mp.
10. (lansoprazole or lanzoprazole or agopton or bamalite or Inhibitol or Levant or Lupizole or lanzor or monolitum or ogast or ogastro or opiren or prevacid or prezal or pro ulco or promeco or takepron or ulpax or zoton).mp.
11. (omeprazole or losec or nexium or prilosec or rapinex or zegerid or ocid or Lomac or omepral or omez).mp.
12. (rabeprazole or aciphex or dexrabeprazole or pariet or zechin or rabecid or nzole-d or rabeloc).mp.
13. (pantoprazole or protium or protonix or pantotab or pantopan or pantozol or pantor or pantoloc or astropan or controloc or pantecta or inipomp or somac or pantodac or zurcal or zentro).mp.
14. exp Histamine H2 Antagonists/
15. ((h2 or histamine) adj2 (blocker$ or agonist$ or receptor$)).mp.
16. (burimamide or cimetidine or famotidine or metiamide or nizatidine or ranitidine).mp.
17. 1 or 2 or 3 or 4 or 5 or 6 or 7 or 8 or 9 or 10 or 11 or 12 or 13 or 14 or 15 or 16

18. exp Critical Illness/
19. exp Critical Care/
20. (critical$ adj2 (ill$ or care)).mp.
21. exp Intensive Care Units/
22. exp Respiration, Artificial/
23. (ICU or intensive care or high dependency unit$ or HDU or intermediate care or mechanical ventilation).mp.
24. exp Neurosurgery/
25. exp Brain/su [Surgery]
26. exp Craniocerebral Trauma/su [Surgery]
27. (neurosurgery or neurosurgical or brain surgery).mp.
28. exp Thorax/su [Surgery]
29. exp Thoracic Surgery/
30. ((cardiothoracic or thorax or thoraracic or chest) adj2 (surgical or surgery or operation)).mp.
31. exp Abdomen/su [Surgery]
32. major abdominal surgery.mp.
33. exp Vascular Diseases/su [Surgery]
34. (vascular adj2 surgery).mp.
35. exp Pelvis/su [Surgery]
36. ((pelvis or pelvic) adj2 surgery).mp.
37. exp Hip/su [Surgery]
38. exp Arthroplasty, Replacement, Hip/
39. (hip adj2 (surgery or replacement or implantation$)).mp.
40. exp Organ Transplantation/
41. ((organ or heart or heart-lung or kidney or liver or lung or pancreas) adj2 transplantation).mp.
42. exp Burns/
43. (burn injury or burn unit or thermal injury).mp.
44. exp Heart Arrest/
45. exp Myocardial Infarction/
46. (coronary care unit or CCU or cardiac intensive care unit CICU or cardiac arrest or cardiac failure or CPR or heart arrest or heart failure or myocardial infarct$).mp.
47. exp Hematologic neoplasms/
48. ((hematologic$ or hematopoietic) adj2 (malignanc$ or neoplasm$ or illness)).mp.
49. exp Acute kidney injury/
50. ((acute kidney or acute renal) adj2 (injur$ or failure or insufficienc$)).mp.
51. exp Liver failure/
52. ((hepatic or liver) adj2 failure).mp.
53. exp Sepsis/
54. sepsis.mp.
55. exp Steroids/dt, tu, th [Drug Therapy, Therapeutic Use, Therapy]
56. (steroid$ adj2 (treatment or therap$)).mp.
57. (high adj2 (dose or dosis)).mp.
58. 55 or 56
59. 57 and 58
60. 18 or 19 or 20 or 21 or 22 or 23 or 24 or 25 or 26 or 27 or 28 or 29 or 30 or 31 or 32 or 33 or 34 or 35 or 36 or 37 or 38 or 39 or 40 or 41 or 42 or 43 or 44 or 45 or 46 or 47 or 48 or 49 or 50 or 51 or 52 or 53 or 54 or 59

61. 17 and 60

62. randomized controlled trial.pt.
63. controlled clinical trial.pt.
64. randomized.ab.
65. placebo.ab.
66. clinical trial.sh.
67. randomly.ab.
68. trial.ti.
69. 62 or 63 or 64 or 65 or 66 or 67 or 68
70. humans.sh.
71. 69 and 70
72. 61 and 71
