# Supplementary material for: Stress ulcer prophylaxis versus placebo or no prophylaxis in adult hospitalised acutely ill patients—protocol for a systematic review with meta-analysis and trial sequential analysis
Source: Syst Rev. 2017 Jun 24;6:118. doi: 10.1186/s13643-017-0509-4 (PMC5483291; doi:10.1186/s13643-017-0509-4)
Supplement: Supplementary file 3 — PRISMA flowchart of study selection. (DOCX 39 kb) [file 13643_2017_509_MOESM3_ESM.docx]

# of records excluded

# of records screened

# of studies included in qualitative synthesis

# of full-text articles excluded, with reasons

# of full-text articles assessed for eligibility

# of records after duplicates removed

# of studies included in quantitative synthesis (meta-analysis)

# of additional records identified through other sources
(n = )

# of non-randomised records database searching
(n = )

# of records identified through database searching
(n = )
